# Flexible Relations Between Confidence and Confidence RTs in Post-Decisional Models of Confidence: A Reply to Chen and Rahnev

**DOI:** 10.1167/jov.24.12.9

**Published:** 2024-11-12

**Authors:** Stef Herregods, Luc Vermeylen, Kobe Desender

**Affiliations:** 1 Brain and Cognition, KU Leuven, Leuven, Belgium; 2 Brain and Cognition, KU Leuven, Leuven, Belgium

**Keywords:** decision confidence, reaction times, metacognition

When making decisions, humans are able to provide well-calibrated estimates of their accuracy, based on their decision confidence (Fleming, Weil, Nagy, Dolan, & Rees, 2010). This ability allows humans to guide subsequent decision-making behavior (Desender, Boldt, & Yeung, 2018; Folke, Jacobsen, Fleming, & De Martino, 2016). Studies investigating the underlying computations of decision confidence frequently point to a post-decisional locus: Post-decisional evidence confirming (contradicting) the decision tends to increase (decrease) decision confidence (Fleming, van der Putten, & Daw, 2018). In line with this, theoretical work suggests that confidence is computed exclusively post-decision (Pleskac & Busemeyer, 2010). It should be noted, however, that there is currently no strong empirical evidence for the claim that confidence is “exclusively” computed post-decision (see Xue, Zheng, Rafiei, & Rahnev, 2023 for evidence to the contrary).

Many theories of decision making assume that humans accumulate noisy perceptual evidence sequentially, until the accumulated evidence reaches a predefined decision boundary (also known as accumulation-to-bound models; see Gold & Shadlen, 2007, for a review). Because the duration of the accumulation of evidence to the decision boundary depends on the strength of the evidence and the height of decision boundaries, such models naturally account for the relationship between decision response times and stimulus difficulty, and for speed-accuracy tradeoffs (e.g., Ratcliff & McKoon, 2008). One common approach to model decision confidence within accumulation-to-bound models is to allow the accumulation process to continue after boundary crossing (Pleskac & Busemeyer, 2010). This extension allows accumulation-to–bound models to explain varying confidence ratings for the same decisions and post-decision error monitoring (Yeung & Summerfield, 2012). When further assuming that the process of post-decisional accumulation continues until reaching a second set of confidence boundaries, it follows that confidence response times (cRTs) should be informative with respect to the process of post-decisional evidence accumulation, and the resulting confidence judgment (Herregods, Denmat, & Desender, 2023; Moran, Teodorescu, & Usher, 2015). In line with this hypothesis, Baranski and Petrusic (1998) already observed consistent, non-monotonic relationships between the level of decision confidence and cRTs across participants.

Recently, Chen and Rahnev (2023) investigated the notion that confidence and cRTs are intrinsically related and showed a wide variety of correlations between confidence and cRTs, with some participants showing a negative relationship (i.e., low confidence associated with long cRTs), but others showing a positive relationship (i.e., high confidence associated with long cRTs). The authors revealed that these individual differences were related to the frequency with which each of the confidence options was used by each participant: Participants tended to have lower cRTs for confidence ratings they chose more often (and vice versa). Furthermore, they stated that “the crucial hypothesis underlying post-decisional evidence accumulation models is that high-confidence responses are inherently made faster” (Chen & Rahnev, 2023, p. 1). Given that our recent post-decision model Herregods et al. (2023) was explicitly mentioned as an example of such a model, we here put this claim to the test by fitting their data with our model. Below, we show that, contrary to the claim made by Chen and Rahnev, the Herregods et al. (2023) model can capture both positive and negative confidence—cRT correlations.

## Modelling confidence ratings and confidence response times

**Figure 1.**
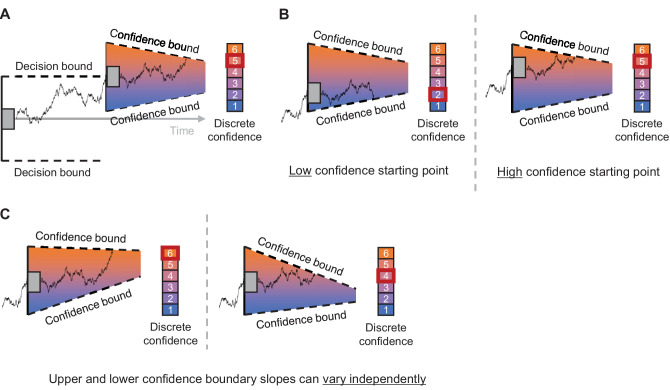
Simplified schematic of the Herregods et al. (2023) n-option model, here shown for six confidence ratings. (**A**) In the Herregods et al. (2023) model, choices are the result of an accumulation-to-bound process. After reaching one of the two choice boundaries, noisy evidence continues to accumulate post-decision, until reaching one of two collapsing confidence boundaries. Post-decision evidence is then linearly mapped onto a discrete six-point confidence scale. (**B**) The *n*-option model variant can capture overall biases in confidence reporting by changing the starting point of the post-decisional accumulation process. The two examples show how a similar trace of post-decision evidence can lead to low versus high confidence depending on the starting point. (**C**) The *n*-option model variant can capture different relationships between confidence and cRTs by separately collapsing the upper or lower confidence boundary as time passes. The two examples show how a similar trace of post-decision evidence can lead to high versus low confidence depending on the confidence boundary collapse rate.

Building on recent post-decision accumulation models of Moran et al. (2015) and Pleskac and Busemeyer (2010), we recently proposed a variant for the computation of decision confidence (Herregods et al., 2023). In Pleskac and Busemeyer (2010), evidence continues to accumulate post-decision after reaching a choice boundary. A critical aspect of our extension is that the accumulation of post-decision evidence terminates once it reaches one of two (potentially collapsing) confidence boundaries (see Figure 1). In Herregods et al. (2023), we developed two model variants: (i) a model variant for binary confidence ratings that predicts high or low confidence depending on the confidence boundary reached, and (ii) a variant for tasks with more fine-grained confidence judgments (i.e., for *n-*option confidence ratings). In the *n-*option variant, the amount of accumulated evidence is linearly mapped onto a confidence value between 1 (i.e., “certainly wrong”) and *n* (“certainly correct”). Similar to how response bias in choices is captured by biasing the starting point of the accumulation process (Ratcliff & McKoon, 2008), for the *n-*option variant we included the possibility that there is a biased starting point for the post-decisional accumulation phase. Finally, this model has separate urgency parameters for the two confidence boundaries (i.e., which control collapse of the confidence boundaries). One reason why participants might use separate levels of urgency for the upper and lower confidence boundary might be to flexibly set optimal parameter combinations in order to maximize reward depending on some externally set reward scheme. A full treatment of optimality, as done by Maniscalco, Charles, and Peters (2022) for signal-detection theory models of confidence, will help to further unravel this point. See Appendix A for a full overview of the model parameters and their interpretation.

To empirically test the claim made by Chen and Rahnev (2023) that our model assumes a negative correlation between confidence and cRTs, we fitted the *n*-option model variant on the same three data sets reported in the main body of their paper. The data was preprocessed the same way as described in Chen and Rahnev (2023), with the following two exceptions to be able to fit the model: (i) Catch trials were removed from the Bang data set, because these trials were significantly easier and implemented to exclude participants with too low performance; (ii) Both the Bang and Haddara1 data sets included data from different experimental conditions, which we here treated as separate participants (i.e., we estimated one set of parameters per experimental condition). Critically, we used exactly the same model and fitting procedure as previously reported and applied in Herregods et al. (2023) Experiment 2 (i.e., the *n-*option variant). The only exception was that for the current analyses we implemented a four-option version of our model because participants reported confidence on a four-point scale, whereas in the original article we used a six-option version. Apart from this, we did not make any further modifications to the model. An overview of all free model parameters can be found in Table A1.

**Figure 2.**
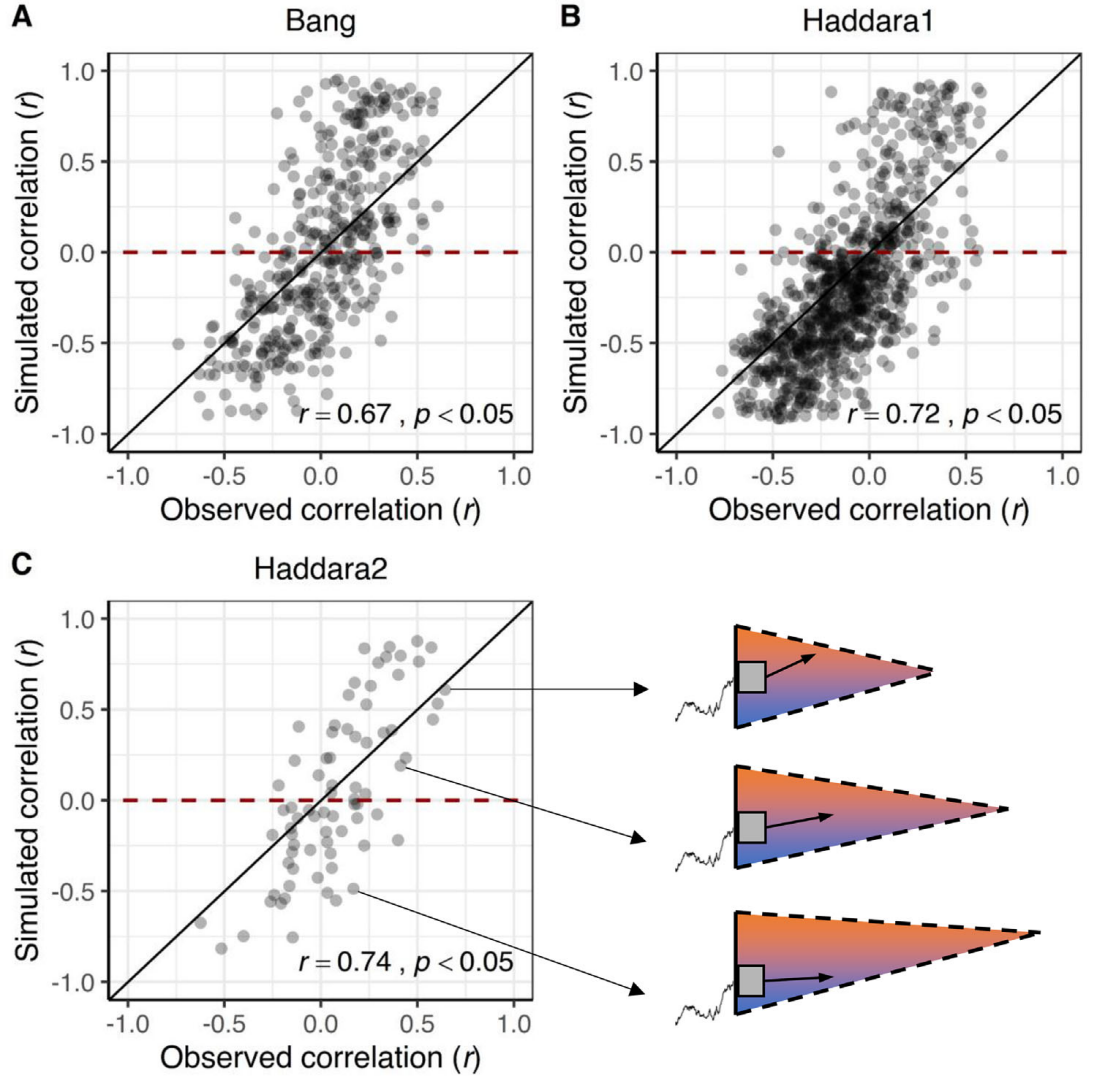
Observed versus predicted correlations between decision confidence and cRTs using the Herregods et al. (2023) n-option variant. Each dot represents data (empirical and model estimate on x and y axis, respectively) from a single participant. The black lines represent the identity lines. These model fits confirm that our model is perfectly able to capture the wide range of correlations between confidence and cRTs reported by Chen and Rahnev (2023), for (**A**) the Bang, (**B**) Haddara1 and (**C**) Haddara2 data sets. To better understand how the model can capture these different patterns, the three figures on the right visualize fitted parameters of the Herregods et al. (2023) model of three example participants.

After fitting the data of all participants using our model, we simulated 1000 trials per participant based on the fitted parameters and then compared the signatures identified by Chen and Rahnev (2023) in the empirical and simulated data. Figure 2 shows the empirically observed Spearman correlations between cRTs and confidence, plotted against the corresponding Spearman correlations predicted by the fitted models. As can be seen, our model provided a good fit to these data*,* capturing the entire range of correlations observed by Chen and Rahnev (2023). To formally test this claim, we showed that in the subset of participants who empirically displayed a negative correlation between confidence and cRTs, we also found significantly negative correlations in model predictions for the Bang (*t*(178) = −9.50, *p* < 0.01), Haddara1 (*t*(588) = −27.58, *p* < 0.01) and Haddara2 (*t*(26) = −4.48, *p* < 0.01) data sets. Vice versa, in the subset of participants who empirically showed a positive correlation between confidence and cRTs, we found significantly positive correlations in model predictions for the Bang (*t*(217) = 10.11, *p* < 0.01), Haddara1 (*t*(276) = 8.07, *p* < 0.01) and Haddara2 (*t*(47) = 4.05, *p* < 0.01) data sets. Note that for these analyses, the Spearman correlations were Fisher *z*-transformed to approximate normal distributions. To understand how our model is able to account for both these patterns, in Figure 2C we visualized the estimated parameters of three example participants showing strongly positive, zero, or strongly negative correlations between confidence and cRTs. As can be seen, the model captured the positive relation by having a high drift rate and a high post-decision starting point, and it captured the negative relation by having a low drift rate and a low post-decision starting point.

**Figure 3.**
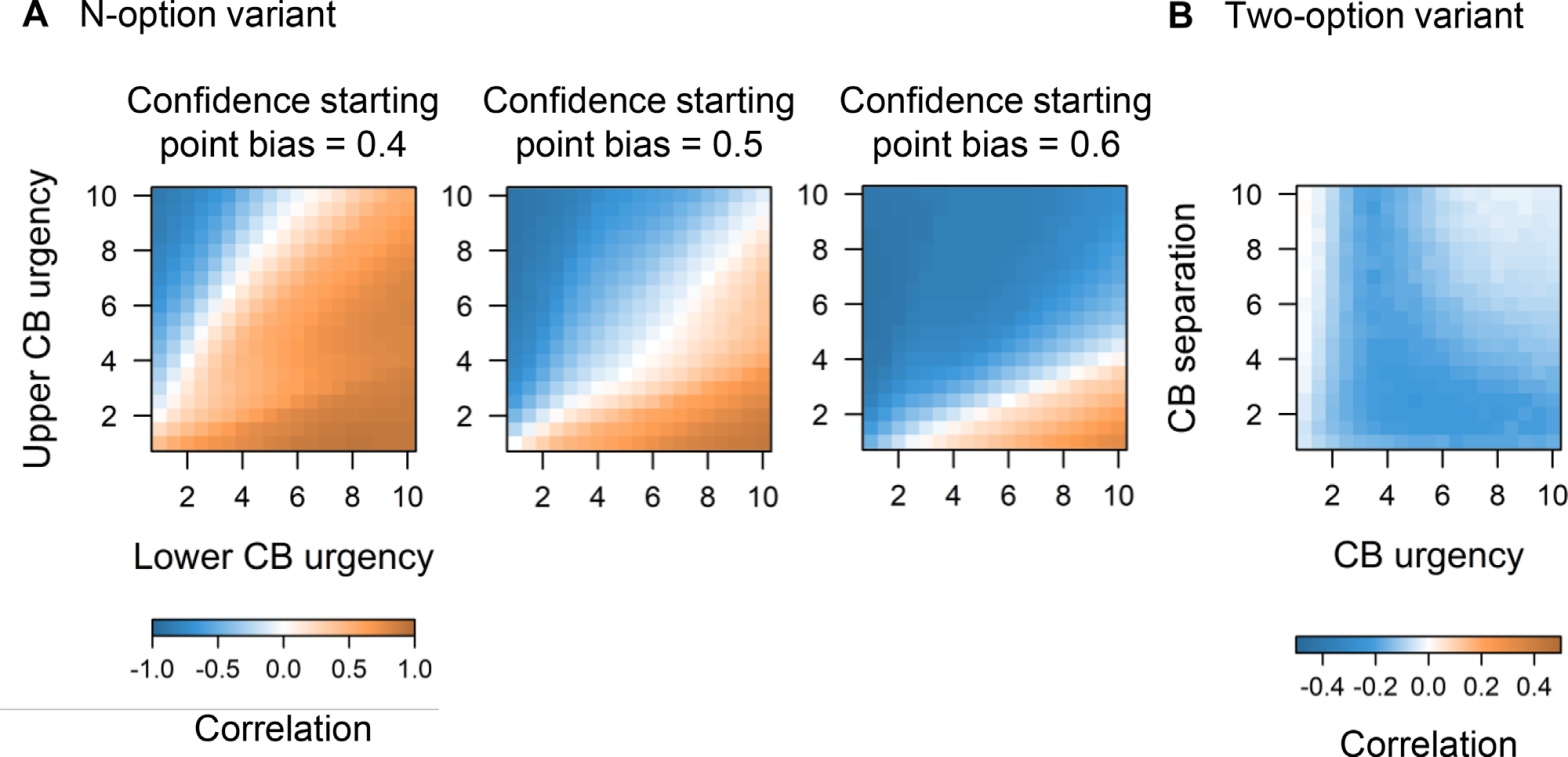
Simulated cj − cRT correlations for the Herregods et al. (2023) *n*-option and two-option variants. CB refers to confidence boundary. The correlation for each set of parameters was computed using 50,000 decision-making trials. (**A**) For the *n*-option variant, the following parameters were fixed: drift rate (1), decision boundary separation (1), confidence boundary separation (5), non-decision time (0), confidence non-decision time (0), *v*-ratio (3). (**B**) For the two-option variant, the following parameters were fixed: drift rate (1), decision boundary separation (1), non-decision time (0), confidence non-decision time (0), *v*-ratio (1).

Going beyond these three example participants, to provide a more complete intuition into how confidence starting point bias and confidence boundary urgencies affect the cj − cRT correlation, we simulated decision-making trials across a wide range of post-decision parameter values. As shown in the heatmaps of Figure 3A, a change in starting point bias and a change in confidence boundary urgencies can independently lead to both positive and negative cj − cRT correlations. In sum, contrary to the claim made by Chen and Rahnev (2023), our model is able to capture both positive and negative correlations between confidence and cRTs.

One important consideration is that in the current reply we only fitted the *n*-option variant of our model to the data, because all experiments reported in Chen and Rahnev (2023) gave participants the option to choose between more than two levels of confidence. Notably, in Herregods et al. (2023) we also described a simpler variant of the model for binary confidence judgments that does not allow for the upper and lower confidence judgment boundary urgencies to vary separately (or for the starting point of post-decision evidence accumulation to be biased). Therefore, given that the drift rate is always positive, this simpler binary confidence variant would not be able to capture positive confidence − cRT correlations as rightfully pointed out by Chen and Rahnev. As can be seen in Figure 3B, the simple model variant can indeed only account for a wide range of positive relations between confidence and confidence RTs.

**Figure 4.**
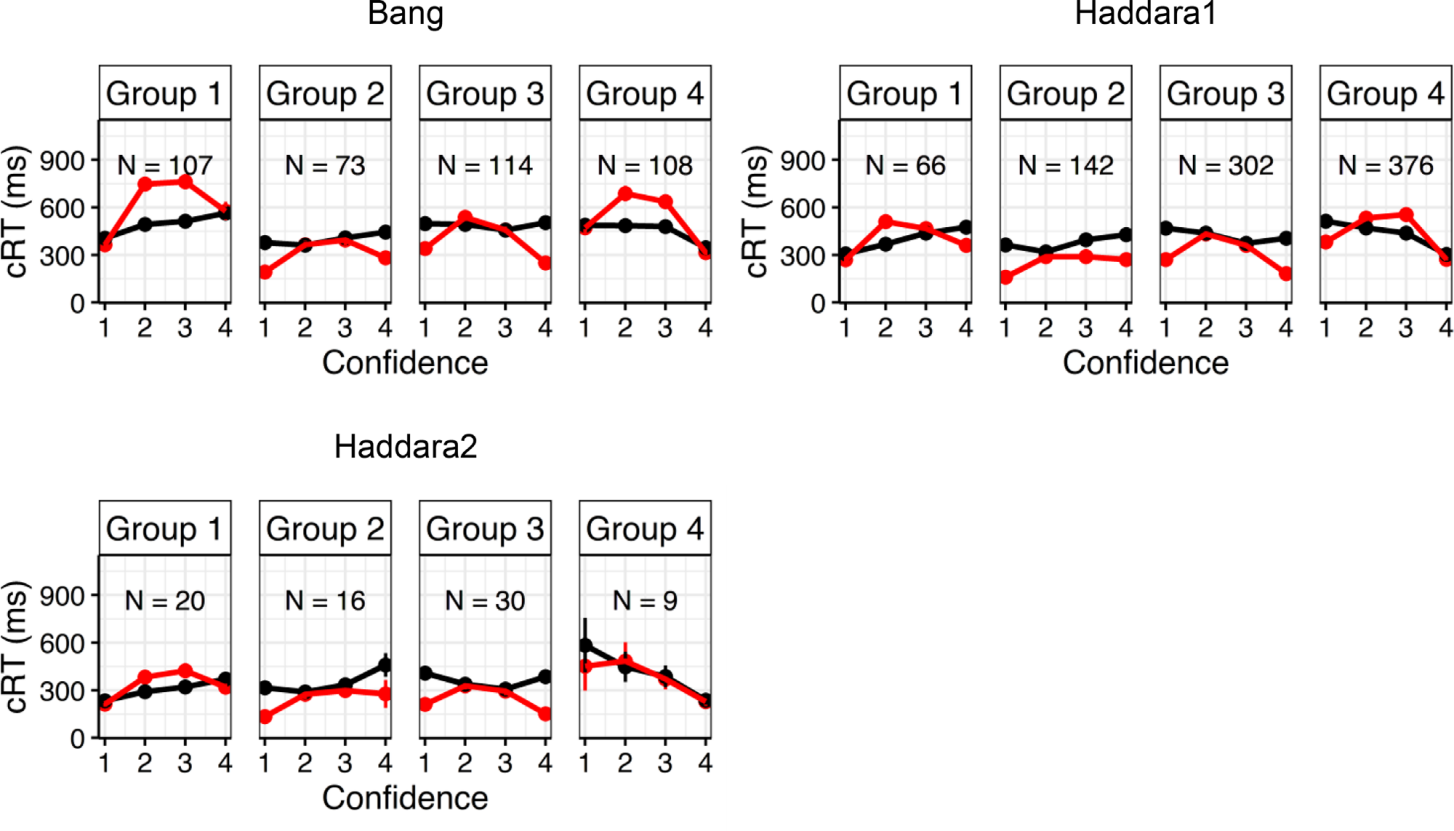
Observed and predicted confidence − cRTs associations. Observed data is shown in black, model predictions in red. Each group consists of all participants that chose the related group number most often as confidence rating.

**Figure B1.**
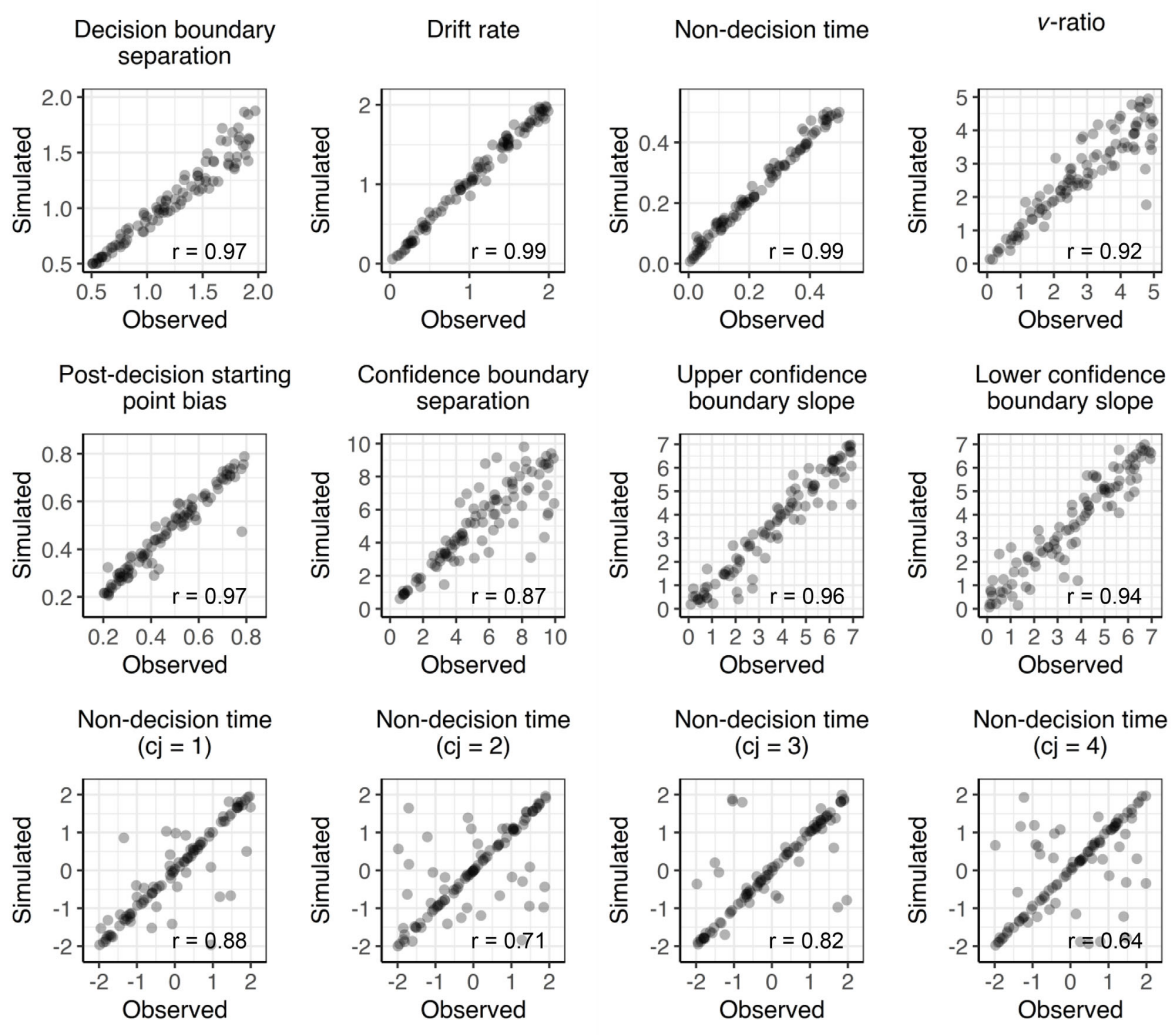
Parameter recovery Herregods et al. (2023) model with confidence rating-specific non-decision times. All 100 sets of parameters were randomly drawn at random from uniform distributions. Reasonable ranges for these distributions were chosen based on previous model fits from the Herregods et al. (2023) *n*-option model variant. From each of these sets of parameters, we generated 10,000 predictions. It can be seen that the main parameters of the Herregods et al. model are still identifiable even when allowing separate non-decision time estimates for each confidence option.

Finally, although this was not the main point of the current investigation, we note that, whereas the Herregods et al. (2023) model can capture both positive and negative correlations between confidence and cRTs, it does seem to fall short in capturing the confidence frequency effect described by Chen and Rahnev (2023). Simulations from the model always predicted that either the lowest or the highest confidence ratings will have the fastest cRT (see Figure 4), not taking into account frequency-based differences in cRT identified by Chen and Rahnev (2023). However, as also suggested by these authors, such differences are likely caused by the motor system being able to execute more frequent actions faster (i.e., similar to how responses made with the dominant hand are usually faster than responses with the non-dominant hand). Thus a straightforward solution to account for this finding would be to allow the model to have separate “motor costs” associated with each confidence option. Notably, the Herregods et al. (2023) model only includes two *non-decision time* parameters capturing non-decision related aspects (such as motor execution time), in choice RTs and cRTs. By further partitioning the non-decision time parameter for confidence responses into confidence option specific estimates (i.e., estimating a motor execution cost per confidence option), it should be possible to also capture these subtle dynamics. Fitting such a model accurately would require a large number of datapoints for each confidence rating. Unfortunately, this requirement does not hold for the datasets analyzed in Chen and Rahnev (2023). For instance, in the Bang data set, participants who most frequently reported a confidence rating of 2 only reported a confidence rating of 4 on 4.67 trials, on average, thus providing insufficient data to estimate separate non-decision time parameters. Nevertheless, to acquire some insight into the possibility of whether robust parameter estimates can be acquired even when estimating non-decision time components separately for each confidence option, we performed a parameter recovery analysis. We randomly sampled 100 sets of parameters from reasonable parameter intervals. As shown in the Figure B1, the added confidence *non-decision time* parameters recovered well, suggesting that at least in theory it should be possible, given the appropriate dataset, to fit such a model to empirical data.

## Conclusions

Chen and Rahnev (2023) emphasize that in empirical data decision confidence can be correlated both positively and negatively with cRTs. In their article, the authors claim that many recent post-decisional models of confidence assume a negative cRT − confidence correlation. Although this claim does indeed hold true for the models proposed in Moran et al. (2015) and Pleskac and Busemeyer (2010), we here we invalidate this claim for the Herregods et al. (2023) *n*-option model variant by fitting their data using our model. By doing so, we showed that our model can capture both positive and negative confidence – cRT correlations.

## Appendix A. Appendix A: Herregods et al. (2023) *n*-option model parameters

**Table A1.** *N*-option model parameters.

Parameter name	Parameter description
Decision boundary separation	Distance between decision boundaries
Drift rate	Average increase of evidence toward the correct decision boundary
Non-decision time	Time during the decision-making process where evidence is not being accumulated (e.g., the time between deciding which button to press and the actual button press because of motor execution time)
Confidence boundary separation	Distance between the confidence boundaries at the start of post-decision evidence accumulation
Confidence starting point bias	Initial bias of evidence toward one of the confidence boundaries at the start of post-decision evidence accumulation
Upper confidence boundary urgency	Speed at which the upper confidence boundary collapses over time
Lower confidence boundary urgency	Speed at which the lower confidence boundary collapses over time
*V*-ratio	Change in drift rate from pre- to post-decision evidence accumulation
Confidence non-decision time	Time during the process of deciding on a confidence level where evidence is not being accumulated (e.g., the time between deciding which button to press and the actual button press because of motor execution time)
